# Freiburg vision test (FrACT): optimal number of trials?

**DOI:** 10.1007/s00417-024-06638-z

**Published:** 2024-09-18

**Authors:** Michael Bach

**Affiliations:** Medical Center – University of Freiburg, Faculty of Medicine, University of Freiburg, Germany, Freiburg, Germany

**Keywords:** Visual acuity, Variability, Efficiency, Precision, Psychophysics, Threshold, LogMAR

## Abstract

**Purpose:**

Visual acuity is a psychophysical threshold that we want to determine as precisely and efficiently as possible. The Freiburg Vision Test FrACT employs the automated Bayesian “Best PEST” algorithm for this purpose: the next optotype size is always selected to be at threshold based on the information acquired so far, thereby maximizing information gain.

**Methods:**

We assessed the test–retest Limits of Agreement (LoA, Bland & Altman 1986) across 6 to 48 trials in 2 × 78 runs involving 26 participants; visual acuity (in part artificially reduced) ranged from 1.22 to -0.59 LogMAR.

**Results:**

LoA exhibited a steep decline from ± 0.46 LogMAR at six trials to ± 0.17 at 18 trials; with more trials, LoA showed less change, reaching ± 0.12 LogMAR at 48 trials. LoA did not significantly change over the wide acuity range assessed here.

**Conclusion:**

These findings suggest that 18 trials represent an efficient balance between precision and burden on the participant and examiner. This observation holds for the eight response alternatives used in this study (8 Landolt C orientations) and is anticipated to apply to the ten Sloan letters as well. With only four choices (e.g., tumbling E), more trials will be necessary.

**Key messages:**

***What is known***

- When assessing visual acuity, a tradeoff between precision and effort is necessary.

***What is new***

- A run length of 18 trials is a good compromise between effort and precision for an 8-alternative task (the Landolt C).
- With 18 trials a 95% confidence interval of ± 0.17 LogMAR for test–retest is found.
- The test–retest precision is independent of the acuity level over the 1.5 LogMAR range studied here.

## Introduction

Visual acuity, the ability to discern fine details and shapes, is a crucial aspect of human vision: Sufficient acuity is essential for reading, writing, and recognizing faces, as well as for many occupations demanding keen visual perception, such as driving, piloting, and performing intricate manual tasks. Moreover, visual acuity is a key indicator of overall eye health, and its assessment is an essential part of routine eye examinations [e.g., 1]. Impaired visual acuity can indicate various eye disorders, such as refractive errors, cataracts, glaucoma, and age-related macular degeneration, all of which can significantly impact an individual's quality of life [2]. Therefore, understanding and monitoring visual acuity is essential to maintain good eye health and ensure timely intervention when vision problems arise.

To assess visual acuity and contrast vision in a semi-automated way, forty  years ago I began to develop the *Freiburg Visual Acuity and Contrast Test* (FrACT) or shorter *Freiburg Vision Test* as a computerized method [3–5]. FrACT has been repeatedly validated and is widely used [see 5 for references]. One particular advantage of FrACT is its wide measurement range [6] which extends from low vision (counting fingers and hand movements [7, 8]) to hyperacuity [9].

A critical property of any acuity test is its precision [10], quantifiable via the test–retest variability. Variability should, of course, be as low as possible (or precision as high as possible), which can be achieved by presenting a large number of optotypes near threshold. This conflicts with the desire for brief/rapid testing (few trials = few presented optotypes) with little burden on the patient’s (or examiner’s) patience and endurance. Given the computerized nature of FrACT, an algorithmically computed stopping criterion would appear a good choice. It could be based on some continuously calculated dispersion measure (as, e.g., in QUEST; [11]). In pilot studies I encountered two drawbacks/ to this, however: (1) Spurious low dispersion measures can occur, by chance, early in the sequence, leading to premature stopping. (2) To estimate dispersion, e.g. by the steepness of the estimated psychometric function, additional trials sufficiently distant from the threshold are required. These add little information to the threshold estimate, but consume testing time.

The aim of this paper is to identify the optimal run length (or number of trials) for FrACT. This was attempted in an early paper of mine (Bach 1996) taking a somewhat idiosyncratic approach. Given the wide use of FrACT since then I deemed it necessary to investigate this question with an established measure for variability. Since the number of trials required for a certain precision depends on the number of optotype alternatives (at least ten for Sloan letters, eight or four for Landolt Cs, four for tumbling Es, two for hyperacuity), we here explore Landolt Cs with eight orientations to find the best compromise between testing effort and precision, with the number of trials ranging from 6 to 48.

## Methods

**Fig. 1:**
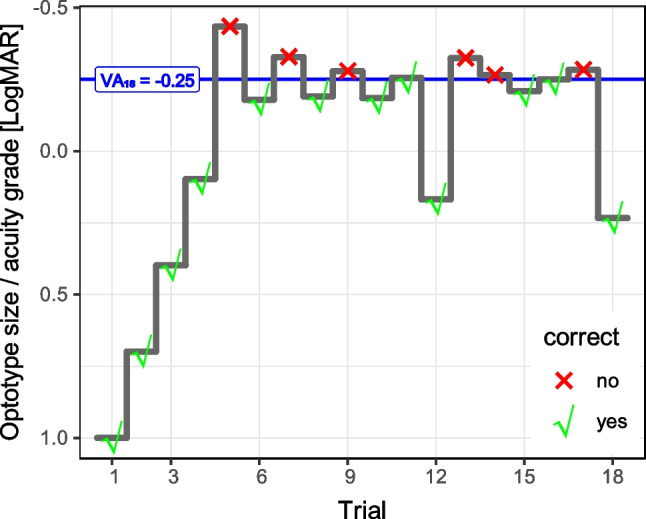
Optotype sizes (corresponding to acuity grades) presented in a typical 18-trial run for an observer with normal acuity (author MB). From a fixed starting size (1.0 LogMAR), the optotype becomes smaller by a factor of two at each trial until an incorrect response (red ×) occurs. Then, the “Best PEST” algorithm takes over, presenting optotypes at the most likely threshold based on information acquired so far. At trials 12 and 18, the optotype is enlarged 3 × to make it “easy”, enhancing motivation, though not much information is gained. The blue horizontal line indicates the final acuity outcome. Note the inverted ordinate: good acuity upward

FrACT uses the automated “Best PEST” algorithm [12] to assess the acuity threshold with an n-alternative forced-choice design: Response data are modeled by a psychometric function with fixed slope, but a variable (and initially unknown) position of its inflection point. The inflection point defines the threshold. Starting from a preset easy-to-see optotype, the next optotype’s size is always chosen to be at threshold based on the information acquired thus far, thus maximizing the information gain (Fig. 1). As a modification to the standard “Best PEST”, the optotype is initially halved in size until the first incorrect response is encountered, then the “Best PEST” algorithm takes over. This avoids too steep a change from the first to the second presentation.

**Fig. 2:**
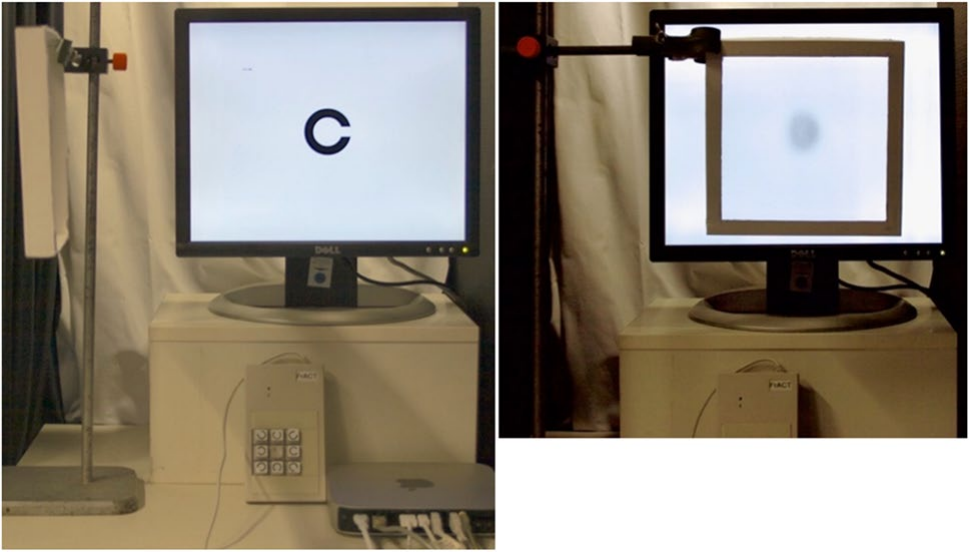
Test setup with the method to reduce effective acuity. Right: the frame with scattering foil nearly obscures the large optotype. Left: the scatter frame is rotated off screen. At the bottom the response pad with its nine buttons is seen, held by the participant during the testing

We employed FrACT version 3.7.1 for the present study. Testing distance was 4 m. To cover a large acuity range, runs with artificially reduced acuity were also included in addition to runs with habitual correction for each eye (OD, OS) and both eyes (OU). To reduce acuity, we applied the “source method”[13], here with a semi-transparent foil taken from office binders. We selected our foil from a variety of transparent office binders with widely different scattering properties to best fit/accommodate our needs (color-neutral, homogeneous, appropriate acuity range). The foil was mounted in a frame and placed at different distances from the screen: When close to the screen, the optical quality was degraded very little; at a distance of a few centimeters the scatter degraded optical quality, easily reducing acuity a factor of ten if desired (Fig. 2). After the first 11 participants it was realized that a wider acuity range was desired, and a second stronger blur condition was added: So 11 participants were examined with habitual acuity and reduced acuity, 15 with an additional, stronger, blur condition. Each condition (participant × eye × blur state) was run twice.

### Participants

After obtaining informed consent from 26 paid participants, we recorded with 3 “eye conditions” (OU, OD, OS). Age ranged from 20 to 59 years with a median of 27 years.

### Analysis

FrACT was set to a run length of n = 48; all trial data were exported after each run for further analysis. To obtain the intermediate acuity results for n = 6, 12, 18, … trials, the presented acuity grade for the n^th^ + 1trial was recoded as acuity outcome. Only data from trials 7, 13, 18… were archived and are available in the accompanying repository  <﻿10.6084/m9.figshare.c.7291279.v2 > .

Raw data were exported from FrACT into a spreadsheet, which was read and further analyzed with R [14] using the “Tidyverse” [15] package. All data and analysis files are included in the repository accompanying this paper. Test–retest variability was quantified via the 95% Limits of Agreement (LoA) metric [16], based on the outcome of the two runs per condition. LoA was calculated with the “blandr” [17] package. To also obtain a measure of dispersion for LoA, I performed bootstraps [18] with 10,000 samples and calculated the empirical quantiles for p = 0.025 and p = 0.975. [For those not wanting to read the source files of the analysis as published in the repository, this is a brief description of the bootstrapping procedure: From all runs with a given trial length, an equal-size subset was drawn, randomized with replacing (thus many runs are duplicated). The LoA of this subset was calculated and stored. This was repeated 10,000 times, yielding an empirical distribution of LoA estimates for that run length].

## Results

**Fig. 3:**
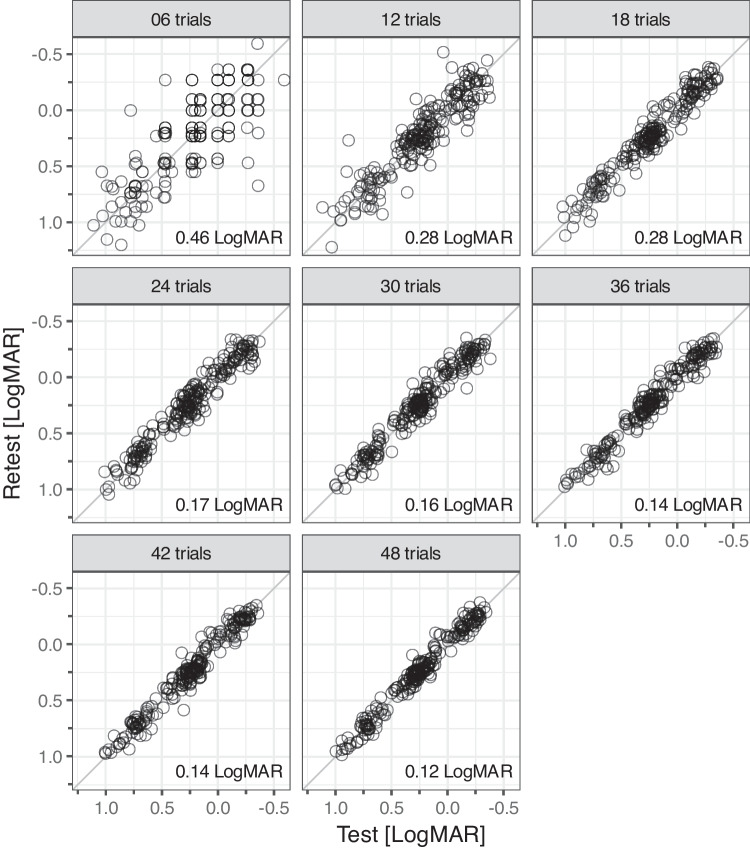
Outcome for all participants (26), eye conditions (3, OU, OD, OS) and run lengths, arranged in test–retest scatter plots segregated by run length (number of trials). Note the inverted axes: good acuity rightward / upward. The bottom right insets indicate the respective limits of agreement

Figure 3 shows all our results in test–retest scatter plots, faceted by run length. An acuity range from 1.22 to -0.59 LogMAR was covered, corresponding to a factor of ≈60 change in visual angle. Without artificial blur and 48-trial runs only, the participants’ acuity ranged from 0. 45 to -0.37 LogMAR.

Data points at or close to the 45-degree identity line indicate low variability (low LoA). Inspection of the figure immediately shows that all data points far away from this line correspond to low trial numbers, as we would expect.

Obviously, short runs often show outliers (points far away from the 45° identity line).

Limits of agreement (LoA) depend on the number of trials.

When there is a marked *bias* (mean test–retest difference) between retest and test, the Limits of Agreement have a differing lower and upper value. Here, this bias was only 0.008 LogMAR, with the 95%-confidence interval ranging from 0.0 to 0.017 LogMAR. A t-test for difference from zero gave a p-value of 0.058. That bias corresponds to two-tenths of a line (with the acuity from the second run being slightly better), and might well be due to chance. Given the low bias, I here simply report the mean LoA Eq. 1:

1 $$LoA={0.5\bullet (LoA}_{upper}-{LoA}_{lower})$$

**Fig. 4:**
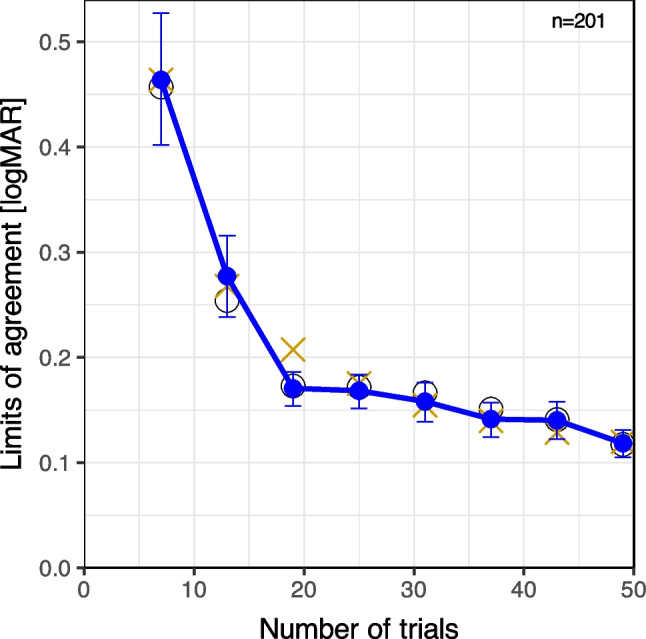
LoAs (test–retest Limits of Agreement) vs. the number of trials. Blue disks with connecting lines and error bars): Standard LoA values with bootstrapped 95% confidence intervals. Blue circles: “Empirical” non-parametric LoAs. The red crosses represent a simple square-root-of-n model as explained in the text

Figure 4 depicts the test–retest limits of agreement (LoA, blue solid line) versus the number of trials. The error bars indicate the bootstrapped 95% confidence intervals. The figure shows that LoA decreases with a growing number of trials (p = 0.015); first steeply from 6 to 18 trials (± 0.47 to ± 0.17 LogMAR), then more slowly to ± 0.12 LogMAR. The shape of the LoA vs. number-of-trials characteristic exhibits a pronounced change in slope (“kink”) at 18 trials (Fig. 4). The test–retest differences from which LoA is calculated was found to be non-normally distributed, there was an excess of outliers. A re-calculation with an “empirical LoA” (non-parametric, based on quantiles, see repository for code) found only slight deviations: the empirical LoA is typically lower by 0.01 LogMAR than the Bland–Altman LoA. This is within symbol size of Fig. 4 and has no effect on interpretation.

A reviewer enquired about theoretical grounds for the run-length findings. So a simple model of the LoA behavior was set up assuming that LoA is mainly governed by the square-root-of-n law, scaling with $1/\sqrt{nTrials}$. This model initially provided a poor fit. Further thought revealed that the first 3 trials are “easy” and nearly always correct, as per default FrACT follows the sequence [LogMAR = 1.0, 0.7, 0.4, 0.1…] until an error occurs (cf. Figure 1). Thus the initial steps just train the participant but do not provide much threshold information; depending on the acuity state the first informative error will occur when a step nears the acuity threshold. So it seemed appropriate to subtract a constant *trialOffset* from the number of trials, and experimentation suggested a *trialOffset* of 3, so ${nTrials}_{eff}=nTrials-trialOffset$. LoA would then scale with run length as follows:

2 $$LoA \sim 1/\sqrt{{nTrials}_{eff}}$$

Applying Eq. (2) and additionally normalizing to coincide at $nTrial=6$ yielded the modeled values indicated by red crosses in Fig. 4. Considering that there are only two free parameters in this simplistic model, namely the *trialOffset* and the overall scaling to let the first pair of points coincide, there is a surprisingly close fit, bringing out all the more the “kink” at 18 trials.

LoA decreases steeply until 18 trials, showing a “kink” (slope change) there, then declines more gradually. Differences between parametric and non-parametric LoAs are of no consequence.

Limits of agreement (LoA) do not depend on the acuity range.

**Fig. 5:**
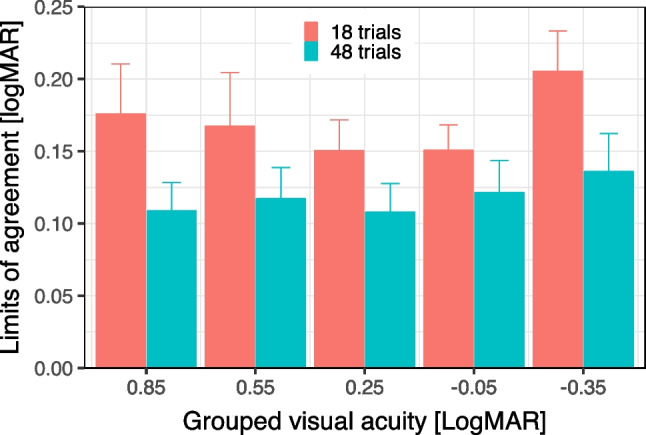
LoA (test–retest Limits of Agreement) versus grouped visual acuity for run lengths of 18 (reddish) and 48 (greenish) trials. Error bars indicate the bootstrapped 95% confidence intervals for LoA

Figure 5 depicts the limits of agreement (LoA) for run lengths of 18 and 48 trials for a number of visual acuity ranges. Acuity (average of test–retest) was binned and grouped in 0.3 LogMAR intervals, and the LoA per group computed. Error bars represent the bootstrapped 95% confidence intervals for LoA. An ANOVA, with factors trialLength and acuityGroup, revealed a significant effect of trialLength (p < 0.01), but not of acuityGroup (p = 0.13) [LoA was normally distributed]. LoA therefore does not seems to depend on visual acuity across the full 1.6-LogMAR-range from 1.13 to –0.47 LogMAR (these values differ from above because here they are derived from the averaged test–retest values).

LoA is lower for 48 than for 18 trials in all acuity ranges while largely independent of acuity within the error margins.

## Discussion

The test–retest variability of visual acuity depends on many factors, including intrinsic threshold noise, observer behavior, number of trials presented, number of choices in a trial (which influences the guessing rate), and the thresholding algorithm used. Here we tried to standardize participant behavior with a forced choice task, with eight choices from a Landolt C, and applied a modified Best-PEST algorithm; the number of trials, i.e., run length, was varied.

I thus assessed test–retest variability of acuity assessment with FrACT over a run length of 48 trials with intermediate results recorded at 6, 12, 18, and so on, up to 48 trials. Variability was quantified with Bland–Altman’s Limits of Agreement (LoA), low values correspond to better reproducibility. Unsurprisingly, LoA improved (i.e., decreased) as the number of trials increased. More surprising was a significant “kink” a 18 trials (Fig. 4). This suggests that 18 trials might represent a good compromise between precision and effort. Numerically this can be expressed by multiplying LoA (to quantify precision) and measurement time; this product exhibits a maximum at 18 trials run length.

Interestingly, LoA did not depend on the participant’s visual acuity, although acuity varied by more than a factor of 60 in visual angle between participants. This complements our findings in a previous assessment of FrACT at ultra-low acuity (Hand Movement and Counting Fingers) [7, 8] where we obtained an LoA of around ± 0.2 (the run length there was 30 trials). Additionally, this also rhymes with recent findings by Britton et al. [personal communication, 19], who report that reproducibility does not suffer even in individuals with very low vision.

When very high precision is desired, one can use longer runs, as would be predicted by model Eq. (2). Alternatively, it might be better to perform two runs of intermediate length instead: This offers the advantage of obtaining a measure of test–retest quality, and the results can be then averaged to improve precision.

Let us discuss possible shortcomings of this design and other error sources:

- The study was designed with a run length of 48 trials, with results for intermediate lengths extracted at trials 6, 12, 18, etc. An alternative design could have involved conducting separate runs for each run length. This approach might have yielded more accurate results, in principle, because the participant's state could differ, for example, at trial 12 when anticipating another 36 trials, compared to trial 12 being the final one. However, such a design would have required eight times more runs. While I lack quantitative data to estimate the influence of a person’s “state” (like vigilance) on acuity results, I suggest that it is low, given that a forced-choice design eliminates a participant’s individual decision criterion (e.g. [20]).
- Could the “kink” be an artifact? The small CI95 error bars (Fig. 4) suggest otherwise. Furthermore, Bach’s [3] somewhat idiosyncratic approach to “deviation from true acuity as a function of [run length]” (1996, Fig. 5) exhibited a similar change in slope derived from a different data set. It remains for future experiments to explore this region and hopefully explain this finding.
- Our participants received payment, which increases motivation compared to patients (especially such as those who have experienced a long waiting period). Considering the rather pronounced change in slope depending on run length and the use of a forced-choice design, I do not expect a marked effect on these findings.

In summary, with 8 choices (i.e., an 8AFC), an 18-trial run length represents an optimal compromise between precision and effort in FrACT. This compromise holds across a wide range of visual acuity levels, resulting in a test–retest limit of agreement of ± 0.17 LogMAR.
